# Smoking Prevalence, Patterns, and Cessation Among Adults in Hebei Province, Central China: Implications From China National Health Survey (CNHS)

**DOI:** 10.3389/fpubh.2020.00177

**Published:** 2020-06-11

**Authors:** Huijing He, Li Pan, Ze Cui, Jixin Sun, Chengdong Yu, Yajing Cao, Ye Wang, Guangliang Shan

**Affiliations:** ^1^Department of Epidemiology and Statistics, Institute of Basic Medical Sciences, Chinese Academy of Medical Sciences and School of Basic Medicine, Peking Union Medical College Beijing, China; ^2^Hebei Provincial Center for Disease Control and Prevention, Shijiazhuang, China

**Keywords:** cigarette smoking, smoking cessation, prevalence, risk factors, alcohol consumption, China

## Abstract

As part of the China National Health Survey, the objective of this study was to explore the prevalence, patterns, and influencing factors of smoking, and understand reasons for smoking cessation among adults in Hebei Province, central China. Using a multi-stage stratified cluster sampling method, 6,552 adults (2,594 males) aged 20–80 were selected in 2017. Demographic, socioeconomic, and tobacco use information were collected by questionnaire interview. The prevalence of ever-smoking, current smoking, and ex-smoking was 28.94, 21.08, and 7.86%, respectively. Male participants had a much higher prevalence of ever-smoking and current smoking (67.39 and 48.77%) than females (3.74 and 2.93%). In male participants, the daily cigarette consumption was 16.61, and the mean age of smoking initiation was 20.95, decreasing with birth year (27.50 in people born before 1946 vs. 17.9 for those born after 1985, *p* for trend < 0.001). Over 40% of male ever-smokers initiated regular smoking before 20. Compared with never drinking, the ORs (95% CI) of ever-smoking for low, moderate, and high alcohol consumption in male participants were 1.44 (1.11–1.86), 2.80 (1.91–4.11), and 2.40 (1.72–3.33), respectively. Among 479 male ex-smokers, 50.94% stopped smoking because of illness and 49.06% by choice. Among male ex-smokers, hypertensive men were more likely to quit smoking than the normotensive individuals (OR: 1.48, 95% CI: 1.13–1.96). For CVD patients, this effect was estimated as 2.27 (95% CI: 1.56–3.30). This study revealed a high prevalence of ever-smoking, especially in men, in a representative population in central China. Health education focus on tobacco control could be integrated with alcohol consumption reduction to achieve additional benefit.

## Introduction

Smoking was the second leading risk factor for early death and disability (1) and is a well-established risk factor for many diseases including hypertension (HTN) (2), diabetes (3), cardiovascular disease (CVD) (4, 5), chronic obstructive pulmonary disease (COPD) (6), and cancer; (7, 8).

The past decade had brought a substantial expansion and strengthening of tobacco control initiatives, such as taxation of tobacco products, bans on smoking in public places and smoke-free zones, etc. (9, 10). However, globally, in 2015, the cigarette smoking prevalence is still high, which is estimated as 25% in men and 5.4% in women (11).

In 2003, the Chinese government signed the WHO Framework Convention on Tobacco Control (FCTC), and efforts have been made in tobacco control (12). Nevertheless, based on the 2015 Chinese Adults Tobacco Survey Report (CATSR) released by the Chinese Center for Disease Control and Prevention (China CDC), about 27.7% (52.1% of men and 2.7% of women) of Chinese population aged 15 and over were current smokers, estimated as over 316 million people, and the average daily tobacco consumption was 15.2 cigarettes (13).

Because of the devastating health, social, economic, and environmental consequences of tobacco consumption (14), understanding the influencing factors of smoking and the reasons for smoking cessation, as well as information on frequency, initiated age, and daily tobacco consumption among smokers, is important for tobacco control in China.

Many factors could be responsible for an individual to initiate and sustain the habit of cigarette smoking. Sociodemographic factors such as gender, educational level, socioeconomic status, and occupation are found to be associated with cigarette smoking (15, 16). As a chronic addictive health-related issue, tobacco dependence is hard to quit that only few smokers managed to quit smoking successfully (17). Since smoking cessation has been shown to have an important effect on health improvement (18, 19), exploring its associated factors is important for tobacco control. However, detailed information about reasons for smoking cessation in Chinese adults was sparse. There were only few publications that focused on general associated factors yielded by regression models, such as sociodemographic characteristics [e.g., job status (20)], health literacy (21) and behaviors [e.g., alcohol intake (22, 23)], or in particular populations such as adolescents (24) or patients with chronic diseases (17, 21).

Hebei Province, located in central China, is one of the study sites of the China National Health Survey (CNHS), and has been added to a large population-based cohort initiated from 2016, with the name of “general population cohort in Beijing, Tianjin and Hebei Provinces” (25). Information collected about smoking and alcohol consumption could provide valuable baseline data and is the basis to estimate the effect of lifestyle risk factors and their effect on health outcomes.

To provide a better understanding of tobacco consumption status and smoking cessation, as well as to provide updated evidence, which CATSR did not provide for health policy makers to develop targeted strategies to promote tobacco control in Chinese population, in this study we attempted to (1) describe the smoking prevalence and smoking patterns, (2) explore associated factors of tobacco consumption and smoking cessation, and (3) understand the reasons for smoking cessation, in a representative population in Hebei Province, central China.

## Methods

### Data Resource and Study Population

The present study was part of the CNHS, which was an ongoing cross-sectional designed population survey conducted from 2012 to 2017 and has been described elsewhere (25). Briefly, we used a multi-stage stratified cluster sampling method to select representative participants. The criteria for participant recruitment were: (1) aged 20–80, and (2) local residency for at least 1 year. The exclusion criteria were: (1) women who were pregnant, (2) soldiers in service, and (3) individuals with severe mental disorders who were not able to finish the questionnaire interview. Data collected in 2017 from Hebei Province (Central China) were used because the detailed information on smoking was collected and it was also baseline data for another new national cohort study.

The sampling procedure consisted of four stages: in the first stage, the provincial capital, one mid-size city, and two counties, one developed and one undeveloped according to the local gross domestic product, were selected; in the second stage, districts were selected from the cities, and rural townships were selected from the counties; in the next stage, streets or communities were selected from districts in urban areas, and villages were selected from towns in rural areas; in the final stage, residents in the selected areas were all invited to participate in the study.

According to CATSR (13), the smoking prevalence in male and female adults in China was 52.1% and 2.7%, respectively. The sample size calculation was based on the following formula:

$$\begin{array}{l} N=\text{Z}\alpha^{2}\times \;pq/d^{2} \end{array}$$

Alpha (α) is the significance level, *p* is the prevalence of smoking, *q* is equal 1 – *p*, and *d* is the error tolerance, which can be estimated as 0.15 × *p*. To reach a significance level of 0.05 and an error tolerance of 0.15*p*, the estimated minimum sample size for male is 157 and for female is 6,004. However, limited by the field work feasibility, finally we recruited 2,594 males and 3,810 females, which had the same proportion of gender and age distribution as the local population.

This study was approved by the institutional review board of the Institute of Basic Medical Sciences, Chinese Academy of Medical Sciences. All participants provided written informed consent.

### Measurements

A structured face-to-face questionnaire interview was administered by trained interviewers. Information collected included the following: (1) demographic and socioeconomic (the highest educational attainment, personal annual income, and occupation) characteristics; (2) personal medical history of HTN, diabetes, and CVD; and (3) lifestyle risk factors such as smoking and alcohol consumption. For ever-smokers, information on the initial age of regular smoking, date of smoking cessation (for ex-smokers only), reasons of smoking cessation (for ex-smokers only), and daily cigarette consumption was collected based on self-report.

#### Assessment of Smoking

Smoking behaviors were measured using self-report of smoking status, intensity of cigarette consumption, smoking duration, and cessation.

Smoking status was categorized into three groups: current smoker, ex-smoker, and never-smoker based on the self-reported amount of smoking and smoking habits (26). Current smoker was defined as smoking at least one cigarette per day (tobacco 1 g/day) and lasting for at least 6 months. Ex-smoker was defined as stopped smoking for more than 6 months preceding the survey. Current smoker and ex-smoker were combined as ever-smoker. Among current smokers and ex-smokers, information about types and amount of tobacco use was collected, with amount calculated in g/day, assuming 1 g of tobacco per cigarette and actual amount in pipes or hand-rolled tobacco as reported. Based on self-reported amount of tobacco use, ever-smoker was further classified into three groups (27): (1) 1–10 cigarettes per day, (2) 11–20 cigarettes per day, and (3) more than 20 cigarettes per day. Among participants who reported smoking cessation, the reasons were recorded and classified into two groups: because of illness or by choice. Smoking duration was derived from the initiate age of regular smoking to age at survey time (current smoker) or age of smoking cessation (ex-smoker).

#### Other Covariates

Current drinkers were defined as those who drank at least twice per month (more than 640 ml of beer or 100 ml of Chinese liquor, about 57 g of alcohol) and had lasted for at least 6 months. Ex-drinkers were defined as those who had stopped drinking more than 6 months prior to the study (28). Within ex-drinker and current drinker, levels of alcohol consumption was defined as low level (30–40 g/day), moderate level (41–60 g/day), and high level (>60 g/day) (29). Educational attainment was categorized into three levels: primary school or lower, middle or high school, and college or higher. Occupation was classified into six groups: (1) farmer, defined as living in rural areas and being engaged in agriculture for a living; (2) factory worker; (3) professional technician, for instance, information technology programmer, engineer, architect, physician, teacher, etc.; (4) officer, such as people working in governmental organizations or associations; (5) unemployed, for example, housewives or husbands who did not have any job; and (6) others, including people who could not be categorized by the above groups, for instance, students or businessmen, etc. The retired participants were classified into the six groups based on their job before retirement. Personal annual income was calculated as (personal monthly income) ×12 or (family annual income)/(number of families) and was classified into four categories: <10,000 RMB/year (1 RMB ≈ 0.14 USD), 10,000–29,999, 30,000–49,999, and 50,000 or above. Self-report history of HTN, diabetes, and CVD (including stroke, intracerebral hemorrhage, coronary heart disease, and myocardial infarction) was recorded. The positive history of the above diseases was defined as ever-diagnosed in an at least township level hospital by a qualified doctor.

### Statistical Analyses

In this study, the analytic sample was restricted to participants with complete information about major risk factors (i.e., age, sex, smoking status). The analyses included 6,552 adults aged 20–80. Eight participants were excluded because of missing value on major risk factors.

Continuous variables were presented as mean ± standard deviation (SD) and categorical data were presented as number (percentage). A *p* < 0.05 (two-tailed) was considered statistically significant. Student's *t*-test for normally distributed data, or Wilcoxon sum-rank test for non-normally distributed data, or chi-square test for categorized data were used to compare characteristics of participants. Cochran-Armitage test was used to perform trend test. General linear regression models (GLMs) were used to perform two-way analysis of covariates (ANCOVA). Multivariable logistic regression models were fitted to explore the associated factors of ever-smoking and smoking cessation, respectively. Because of limited numbers of female ever-smokers, the analyses on influencing factors of ever-smoking, smoking cessation, and reasons for smoking cessation were restricted to male participants.

All statistical analyses were performed using SAS 9.4 (SAS Institute Inc. Cary, NC, USA).

## Results

### Demographic Characteristics and Smoking Prevalence

The analyses for this study were based on 6,552 adults (2,594 males) aged 20–80 with an average age of 48.26 ± 13.63. The demographic characteristics and smoking prevalence are shown in Table 1.

**Table 1:** Characteristics of participants in Hebei Province of China, stratified by smoking status, 2017.

	**Male**						**Female**					
**Characteristics**	**Total**		**Never-smoker**		**Ever-smoker**		**Total**		**Never-smoker**		**Ever-smoker**	
	**(*****n*** **=** **2,594)**		**(*****n*** **=** **846)**		**(*****n*** **=** **1,748)**		**(*****n*** **=** **3,958)**		**(*****n*** **=** **3,810)**		**(*****n*** **=** **148)**	
	***n***	**%**	***n***	**%**	***n***	**%**	***n***	**%**	***n***	**%**	***n***	**%**
**Age group**												
20–29	318	12.26	112	13.24	206	11.78	494	12.48	489	12.83	5	3.38
30–39	433	16.69	164	19.39	269	15.39	716	18.09	701	18.40	15	10.14
40–49	526	20.28	207	24.47	319	18.25	956	24.15	931	24.44	25	16.89
50–59	698	26.91	203	24.00	495	28.32	990	25.01	950	24.93	40	27.03
60–80	619	23.86	160	18.91	459	26.26	802	20.26	739	19.40	63	42.57
p^*^	<0.001						<0.001					
*p* for trend	<0.001						<0.001					
**Resident area**^**&**^												
Urban	1,090	42.02	416	49.17	674	38.56	1,879	47.47	1,850	48.56	29	19.59
Rural	1,503	57.94	429	50.71	1,074	61.44	2,072	52.35	1,954	51.29	118	79.73
p^*^	<0.001						<0.001					
**Education attainment**^**&**^												
Primary school or lower	564	21.74	114	13.48	450	25.74	1,433	36.21	1,327	34.83	106	71.62
High school	1,488	57.36	464	54.85	1,024	58.58	1,714	43.30	1,678	44.04	36	24.32
College or higher	541	20.86	267	31.56	274	15.68	807	20.39	804	21.10	3	2.03
p^*^	<0.001						<0.001					
*p* for trend	<0.001						<0.001					
**Occupation**^**&**^												
Farmer	395	15.23	99	11.70	296	16.93	666	16.83	640	16.80	26	17.57
Factory worker	161	6.21	41	4.85	120	6.86	185	4.67	179	4.70	6	4.05
Technician	400	15.42	176	20.80	224	12.81	747	18.87	746	19.58	1	0.68
Officer	133	5.13	65	7.68	68	3.89	92	2.32	91	2.39	1	0.68
Unemployed	157	6.05	41	4.85	116	6.64	1,076	27.19	993	26.06	83	56.08
Others	1,348	51.97	424	50.12	924	52.86	1,190	30.07	1,159	30.42	31	20.95
p^*^	<0.001						<0.001					
**Personal income (RMB/year)** ^**&**^												
<10,000	591	22.78	148	17.49	443	25.34	1,059	26.76	991	26.01	68	45.95
10,000–29,999	753	29.03	223	26.36	530	30.32	1,627	41.11	1,575	41.34	52	35.14
30,000–49,999	754	29.07	294	34.75	460	26.32	969	24.48	952	24.99	17	11.49
≥50,000	491	18.93	180	21.28	311	17.79	275	6.95	267	7.01	8	5.41
p^*^	<0.001						<0.001					
*p* for trend	<0.001						<0.001					
**Alcohol drinking**^**&**^												
Never drinking	305	11.76	125	14.78	180	10.30	2,819	71.22	2,735	71.78	84	56.76
Quit drinking	254	9.79	67	7.92	187	10.70	159	4.02	147	3.86	12	8.11
Current drinking	2,035	78.45	654	77.30	1,381	79.00	978	24.71	926	24.30	52	35.14
p^*^	<0.001						<0.001					
**Alcohol consumption (g/day)**^**&**^												
Never drinking	305	11.76	125	14.78	180	10.30	2,819	71.22	2,735	71.78	84	56.76
<40	1,576	60.76	560	66.19	1,016	58.12	1,066	26.93	1,018	26.72	48	32.43
41–60	273	10.52	58	6.86	215	12.30	40	1.01	32	0.84	8	5.41
>60	440	16.96	103	12.17	337	19.28	29	0.73	21	0.55	8	5.41
p^*^	<0.001						<0.001					
*p* for trend	<0.001						<0.001					

* Comparisons between never-smoker and ever-smoker in the same sex;

& *The sum of the proportion was not equal to 100% because of missing values. 1 RMB ≈ 0.14 USD*.

**Figure 1:**
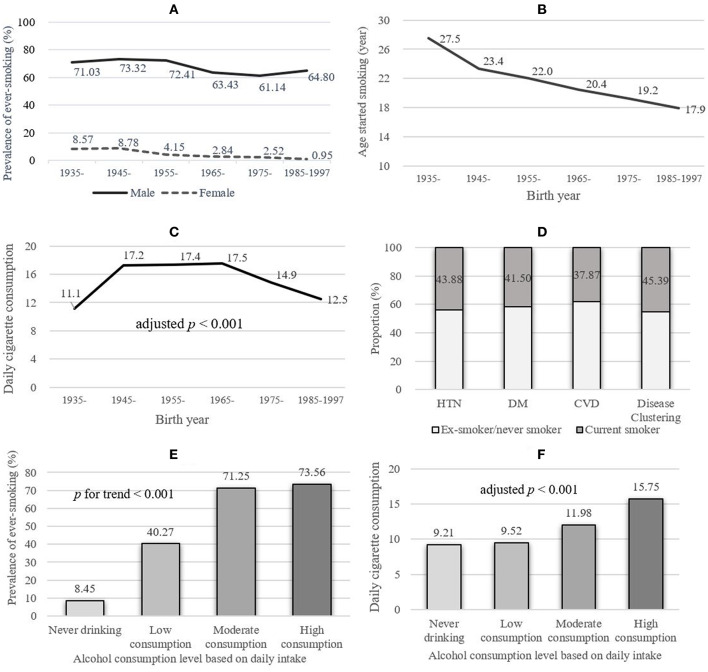
The smoking prevalence and patterns among participants aged 20–80 in Hebei Province, central China, 2017. HTN, hypertension; DM, diabetes; CVD, cardiovascular disease; disease clustering: having more than one disease of HTN, DM, or CVD. Alcohol consumption categories: low (<40 g/day), moderate (41–60 g/day), and high (>60 g/day). Cochran–Armitage test was used to perform trend test. **(A)** Sex- and age-specific prevalence of ever-smoking. **(B)** The education-adjusted regular smoking started age among male ever-smokers, stratified by different birth year groups. **(C)** Daily cigarette consumption of male ever-smokers, stratified by different birth year groups. **(D)** The prevalence of current smoking among male participants with diagnosed chronic diseases. **(E)** The prevalence of ever-smoking stratified by alcohol consumption levels. **(F)** Age- and education-adjusted daily cigarette consumption of male ever-smokers, stratified by alcohol consumption.

The prevalence of ever-smoking, currenting smoking, and ex-smoking was 28.94, 21.08, and 7.86%, respectively. Male participants had a much higher prevalence of ever-smoking and current smoking (67.39 and 48.77%) than females (3.74 and 2.93%). To better illustrate the time trend of smoking prevalence, sex-specific ever-smoking prevalence classified by birth-year groups was calculated and shown in Figure 1A. For both sexes, the prevalence of ever-smoking decreased with time (both *p* < 0.001).

Compared with participants from urban areas, rural subjects had higher prevalence of ever-smoking (male: 71.46 vs. 61.83%, *p* < 0.001; female: 5.69 vs. 1.54%, *p* < 0.001) in both sexes. Notably, an increasing trend of ever-smoking prevalence with elevated alcohol consumption level was observed, from 8.45% in the never drinking group to 73.56% in the high consumption group (*p* < 0.001, Figure 1E).

### Smoking Patterns

The smoking patterns of male participants are shown in Table 2. Nearly all male current smokers (97.0%) used manufactured cigarettes only. Among male smokers, the average age of start smoking regularly was 20.95, decreasing steadily with time (27.5 in those born in 1935–1945 vs. 17.9 in 1985–1997, adjusted for education, *p* < 0.001, see Figure 1B). The average smoking duration in ever-smokers was 25.69 years. The daily cigarette consumption among birth groups was presented in Figure 1C.

**Table 2:** Smoking patterns, stratified by smoking status among male subjects in Hebei Province, central China, 2017.

	**Ever-smoker**		**Current smoker**		**Ex-smoker**		**Cessation because of**		**Cessation by choice**	
	**(*****n*** **=** **1,748)**		**(*****n*** **=** **1,265)**		**(*****n*** **=** **483)**		**illness (*****n*** **=** **244)**		**(*****n*** **=** **235)**	
**Smoking started age** **(year, mean, SD)**	20.95	6.25	21.39	7.03	21.78^a^	6.59	22.03	6.29	21.56	6.93
**Categories (*****n*****, %)**										
<15	98	5.61	84	6.64	14	2.90^a^	3	1.23	11	4.68
15–19	634	36.27	471	37.23	163	33.75	79	32.38	83	35.32
20–29	844	48.28	590	46.64	254	52.59	134	54.92	117	49.79
>30	172	9.84	120	9.49	52	10.77	28	11.48	24	10.21
**Smoking duration** (years, mean, SD)	25.69	13.10	26.58	13.36	23.01^a^	13.00	26.49	12.75	19.55	12.30
**Categories (*****n*****, %)**										
<5	68	3.89	35	2.77	33	6.83^a^	11	4.51	21	8.94^b^
5–9	141	8.07	101	7.98	40	8.28	11	4.51	28	11.91
10–19	392	22.43	272	21.50	120	24.84	48	19.67	70	29.79
20–29	424	24.26	306	24.19	118	24.43	59	24.18	59	25.11
>30	718	41.08	551	43.56	167	34.58	111	45.49	56	23.83
**Daily cigarette** **consumption** **(mean, SD)**	16.61	10.72	15.95	9.99	16.81	12.83	18.11	12.90	15.48	12.70
**Categories (*****n*****, %)**										
≤ 10	749	42.85	218	45.13	531	41.98	96	39.34	120	51.06^b^
11–19	772	44.16	197	40.79	575	45.45	108	44.26	87	37.02
>20	227	12.99	68	14.08	159	12.57	40	16.39	28	11.91
**Duration from quit smoking to current time** (years, mean, SD)	NA	NA	NA	NA	12.05	10.15	10.81	9.54	13.25	10.46
**Categories (n, %)**										
<2	NA	NA	NA	NA	36	7.45	20	8.20	16	6.81^b^
2–4	NA	NA	NA	NA	107	22.15	62	25.41	44	18.72
5–9	NA	NA	NA	NA	81	16.77	47	19.26	34	14.47
≥10	NA	NA	NA	NA	245	50.72	107	43.85	135	57.45

a p < 0.01 of the comparison between current smoker and ex-smoker;

b *p < 0.05 of the comparison between people quit smoking because of illness and people quit smoking by choice. The sums of the proportion in some columns were not equal to 100% because of missing values. NA, not applicable*.

42.90% of ever-smokers initiated regular smoking at an age younger than 20. Among ever-smokers, over 65% had a smoking duration more than 20 years, of which 62.87% smoked for more than 30 years. 57.15% of ever-smokers smoked more than 10 cigarettes per day. For ex-smokers, 89.64% had been stopped for no <2 years (Table 2).

Among 2,594 male participants, 892 (34.39%) were ever diagnosed with HTN, 253 (9.75%) diagnosed with diabetes, and 272 (10.49%) for CVD. The current smoking prevalence among participants ever being diagnosed with HTN, diabetes, and CVD was 43.88, 41.50, and 37.87%, respectively (Figure 1D and Table S2).

Among ever-diagnosed HTN patients, younger people, i.e., at the age of 20–29, had the highest self-reported current smoking prevalence (62.37%). The prevalence of current smoking among ever-diagnosed HTN patients increased with alcohol consumption, from 36.67 to 49.49% (*p* for trend < 0.01). Among ever-diagnosed diabetes patients, an increasing prevalence of current smoking along with elevated alcohol consumption was also observed (*p* for trend < 0.01). Among CVD patients, younger people, moderate, or high-level alcohol consumption had higher current smoking prevalence, but the differences were not statistically significant (Table S2).

The smoking patterns and smoking prevalence among ever-diagnosed chronic disease patients of female smokers were presented in Supplemental Materials (Tables S1, S2). Because of the limited sample size of female smokers (*n* = 148), we did not perform statistical tests.

### Factors Associated With Ever-Smoking

The influencing factors of ever-smoking among male participants are shown in Table 3. Men reporting lower educational attainment were more likely to smoke, *p* for trend <0.001. Compared with subjects who reported college or higher education, the odds ratios (95% CI) for participants who only finished primary school or lower, and middle or high school were 3.07 (2.16–4.36) and 1.84 (1.42–2.39), respectively. Alcohol consumption had a positive effect on smoking in all consumption levels. Compared with never drinking, the ORs (95% CI) for low, moderate, and high alcohol consumption levels were 1.44 (1.11–1.86), 2.80 (1.91–4.11), and 2.40 (1.72–3.33), respectively. The factors influencing ever smoking stratified by urban-rural areas were presented in Table S3. To further analyze the effect of alcohol consumption on tobacco use, the age- and education-adjusted means of daily cigarette consumption were presented and compared (see Figure 1F). The daily average cigarette amount increased with elevated alcohol consumption levels, from 9.21 per day in the never drinking group to 15.75 per day in the high alcohol consumption group (*p* < 0.001). Participants in rural areas were more likely to smoke. However, after adjusting for other covariates, the odds ratio lost its statistical significance. After the adjustment of covariates, occupation and income were not found to be associated with ever-smoking.

**Table 3:** The associated factors of ever-smoking in male participants in Hebei Province, central China, 2017.

	***n***	**%**	**C-OR**	**95% CI**		***p***	**A-OR**	**95% CI**		***p***
**Age** (every 10 years)	NA	NA	1.16	1.09	1.23	<0.001	1.06	0.99	1.14	0.094
**Resident area**										
Urban	1,090	42.02	1	NA	NA	NA	1	NA	NA	NA
Rural	1,503	57.94	1.55	1.31	1.82	<0.001	1.06	0.86	1.31	0.573
**Education attainment**										
Primary school or lower	564	21.74	3.85	2.95	5.02	<0.001	3.07	2.16	4.36	<0.001
High school	1,488	57.36	2.15	1.76	2.63	<0.001	1.84	1.42	2.39	<0.001
College or higher	541	20.86	1	NA	NA	NA	1	NA	NA	NA
**Occupation**										
Farmer	395	15.23	1	NA	NA	NA	1	NA	NA	NA
Factory worker	161	6.21	0.98	0.64	1.49	0.921	1.27	0.79	2.07	0.327
Technician	400	15.42	0.43	0.32	0.58	<0.001	0.89	0.61	1.32	0.568
Officer	133	5.13	0.35	0.23	0.53	<0.001	0.70	0.43	1.15	0.158
Unemployed	157	6.05	0.95	0.62	1.44	0.798	1.01	0.65	1.58	0.969
Others	1,348	51.97	0.73	0.57	0.94	0.015	1.03	0.75	1.40	0.869
**Personal income (RMB per year)**^**a**^										
<10,000	591	22.78	1	NA	NA	NA	1	NA	NA	NA
10,000–29,999	753	29.03	0.79	0.62	1.01	0.063	0.98	0.74	1.30	0.886
30,000–49,999	754	29.07	0.52	0.41	0.66	<0.001	0.87	0.64	1.19	0.389
≥50,000	491	18.93	0.58	0.45	0.75	<0.001	1.02	0.73	1.44	0.889
**Alcohol consumption (g/day)**										
Never drinking	305	11.76	1	NA	NA	NA	1	NA	NA	NA
<40	1,576	60.76	1.26	0.98	1.62	0.071	1.44	1.11	1.86	0.007
41–60	273	10.52	2.57	1.78	3.72	<0.001	2.80	1.91	4.11	<0.001
>60	440	16.96	2.27	1.65	3.12	<0.001	2.40	1.72	3.33	<0.001

a *1 RMB ≈ 0.14 USD. A-OR, adjusted odds ratio; CI, confidence interval; C-OR, crude odds ratio; NA, not applicable*.

### Influencing Factors and Reasons of Smoking Cessation

Being diagnosed with chronic disease was positively associated with smoking cessation among male ever-smokers. Hypertensive males were more likely to quit smoking than their counterparts (OR: 1.48, 95% CI: 1.13–1.96). For CVD patients, this effect was estimated as 2.27 (95% CI: 1.56–3.30). People who had ever been diagnosed with diabetes were not observed to be associated with smoking cessation (OR: 0.96, 95% CI: 0.63–1.47, *p* = 0.853).

It is more difficult for smokers who had longer tobacco consumption time to quit smoking, but heavy smokers were more likely to stop smoke, yet with a marginal OR (1.01–2.26) and *p*-value (0.047). Compared with men with college or above educational level, the primary school or below group was more likely to quit smoke; the OR (95% CI) was 1.71 (1.05–2.78) (Table 4).

**Table 4:** The associated factors of smoking cessation among male ever-smokers in Hebei Province, central China, 2017.

	***n***	**%^**a**^**	**C-OR**	**95% CI**		***p***	**A-OR**	**95% CI**		***p***
**Age** (every 10 years)	NA	NA	1.81	1.64	1.99	<0.001	4.47	3.73	5.36	<0.001
**Resident area**										
Urban	674	28.19	1.00	NA	NA	NA	1.00	NA	NA	NA
Rural	1,074	27.28	0.96	0.77	1.185	0.679	0.84	0.62	1.13	0.246
**Education attainment**										
Primary school or lower	450	32.00	1.51	1.08	2.13	0.018	1.71	1.05	2.78	0.030
High school	1,024	26.76	1.18	0.86	1.60	0.310	1.13	0.75	1.70	0.574
College or higher	274	23.72	1.00	NA	NA	NA	1.00	NA	NA	NA
**Alcohol consumption (g/day)**										
Never drinking	180	21.67	1.00	NA	NA	NA	1.00	NA	NA	NA
<40	1,016	28.44	1.44	0.98	2.10	0.061	1.42	0.91	2.24	0.126
41–60	215	29.30	1.50	0.95	2.37	0.085	1.00	0.57	1.74	0.990
>60	337	27.30	1.36	0.89	2.08	0.162	1.26	0.76	2.11	0.370
**Diagnosed HTN**										
Yes	633	38.23	2.15	1.73	2.68	<0.001	1.48	1.13	1.96	0.005
No	998	22.34	1.00	NA	NA	NA	1.00	NA	NA	NA
**Diagnosed DM**										
Yes	167	29.78	1.39	0.99	1.96	0.058	0.96	0.63	1.47	0.853
No	1,004	37.13	1.00	NA	NA	NA	1.00	NA	NA	NA
**Diagnosed CVD**										
Yes	192	46.35	2.55	1.88	3.46	<0.001	2.27	1.56	3.30	<0.001
No	1,556	25.32	1.00	NA	NA	NA	1.00	NA	NA	NA
**Smoking duration (years)**										
<5	35	48.53	1.00	NA	NA	NA	1.00	NA	NA	NA
5–9	40	28.37	0.42	0.23	0.77	0.004	0.61	0.26	1.43	0.252
10–19	120	30.61	0.47	0.28	0.79	0.004	0.31	0.15	0.65	0.002
20–29	118	27.83	0.41	0.24	0.69	<0.001	0.05	0.02	0.12	<0.001
>30	167	23.26	0.32	0.19	0.53	<0.001	0.01	0.00	0.02	<0.001
**Daily cigarette consumptions**										
≤10	218	29.11	1.00	NA	NA	NA	1.00	NA	NA	NA
11–19	197	25.52	0.84	0.67	1.05	0.117	1.00	0.76	1.33	0.977
>20	68	29.96	1.04	0.75	1.44	0.805	1.51	1.01	2.26	0.047

a *Prevalence of smoking cessation. A-OR, adjusted odds ratio; CI, confidense interval; C-OR, crude odds ratio; CVD, cardiovascular disease; DM, diabetes; HTN, hypertension*.

**Figure 2:**
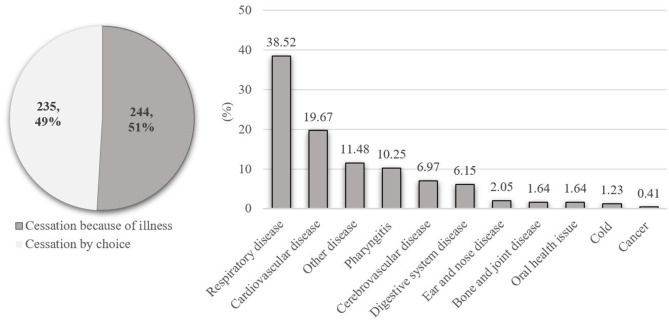
Reasons for smoking cessation among male ex-smokers aged 20–80 in Hebei Province, central China, 2017.

Reasons for smoking cessation were classified into two groups: because of illness and by choice. Among 483 male ex-smokers, 479 reported reasons for smoking cessation, of whom 244 (50.94%) stopped because of illness and the other 235 (49.06%) stopped by choice. For people who stopped smoking because of illness, respiratory disease (94, 38.52%) ranked the top and CVD ranked the second (48, 19.67%). The frequency and percentage of other diseases were presented in Figure 2 and Table S4. Among people stopped by choice, three stopped because of spousal pregnancy, three because of economic pressure, and two stopped because of the initiation of military service. A total of 227 out of 235 men who stopped smoking by choice did not report specific reason. The basic characteristics of men who stopped smoking because of illness and by choice were presented and compared in Table S5. Male ex-smokers who stopped smoking because of illness were older than their counterparts (*p* < 0.001).

## Discussion

Using a well-designed sampling method, we collected a representative sample of adult participants living in Hebei Province, central China. The present survey revealed a high prevalence of smoking in the study population, especially in men (67.39%), and explored the influencing factors and main reasons for smoking cessation, which may provide useful evidence for policy makers to initiate more targeted and effective interventions to curb tobacco use.

The adoption of FCTC, together with other tobacco control programs, has brought a wide range of effective interventions and policy instruments for tobacco epidemic (10, 30). However, the pace of progress in reducing smoking prevalence has been heterogeneous across geographies and development status (11). Much of the increase in tobacco use and the associated morbidity and mortality occur in low- and middle-income countries (31). Revealed by the Global Burden of Disease Study, China, India, and Indonesia have the largest number of smokers, which accounted for 51.4% of the world's male smokers in 2015 (11).

Considering the great number of smokers and large health hazard attributable to smoking, the Chinese government has adopted many interventions to curb tobacco use. There are mainly four tobacco control policy interventions in China: the international public health treaty, FCTC by WHO; the government's national policy forbidding using general revenue to purchase cigarettes, and smoking in public by state employees or officials; and the tax increases in 2009 and 2015, respectively (12, 32). Despite these efforts, the prevalence of smoking was still high. The Global Adults Tobacco Survey (GATS) showed that 52.9% Chinese men were current smokers in 2010, ranked the second among the 16 countries that completed the survey (33). A national survey, China Non-communicable Disease Surveillance, showed that 54.1% of men and 2.6% of women were current smokers in 2011 (34). Data derived from the China Health and Nutrition Survey presented a slightly decreased, but still high prevalence of smoking in Chinese men, from 60.6% in 1991 to 51.6% in 2011. A system review reported that in 2015, the prevalence of daily smoking in China was 37.5% for men and 2.2% for women (11).

According to CATSR, the current smoking prevalence was 51.4% in men and 4.1% in women in Hebei Province located in central China, and the current smoking prevalence investigated by this survey was similar to what was found in CATSR, which was 48.77% in men and 2.93% in women. Female smoking prevalence is dramatically lower than males, and this is a common phenomenon in developing countries (35). This prevalence disparity may be due to gender inequality, higher total fertility rates, and better awareness of health risks related to active smoking (35, 36).

In line with other studies, people from rural areas, with lower educational attainment, had higher prevalence of current smoking (33). In developing countries, lower SES was associated with higher risk of smoking behavior (37). Similar with Wang's study (37), people with lower educational level were more likely to smoke. Education may reflect an individual's health-related knowledge and ability to making health-conscious decisions such as tobacco use (38). In this study, personal annual income lost its statistical significance in association with smoking after other covariates were adjusted. Occupation was not found to be associated with smoking among male participants. Supported by the regression models, the urban–rural smoking prevalence disparity may be attributed to the urban–rural difference in educational attainment. Besides, urban–rural difference on psychosocial characteristics may also partially explain this disparity (39).

In 2015, alcohol consumption and tobacco use were estimated to cost the human population more than a quarter of a billion disability (40). Though alcohol consumption and tobacco use are each risk factors for disease, they are highly co-morbid, and the consequences of concurrent use are multiplicative (41). Previous studies supported that people who drink alcohol were more likely to smoke (42, 43) and there is a positive association between alcohol consumption and number of cigarettes smoked (44). Similarly, in this study, both ever and current smoking prevalence increased along with elevated alcohol consumption dose, and the daily cigarette consumption also increased with alcohol consumption. This positive relationship was also found in the risk factors assessment, where heavy drinkers were more likely to smoke. A review on alcohol–tobacco interactions in human laboratory concluded that alcohol and tobacco have reciprocal influences on potential craving, subjective responses to fixed-dose alcohol or nicotine administration, and self-administration (41). The mechanisms of this association may include genes involved in regulating neurotransmitters, cross-tolerance, and sensitization to both drugs and common social and psychological factors (45, 46). The association between alcohol consumption and smoking found in this study suggests that policies that aim to reduce alcohol drinking may have additional benefit of reducing tobacco consumption or vice versa.

When smoking patterns were examined further, we found that the mean age of smoking initiation declined in the male ever-smokers, from 27.5 among people born before 1945 to 17.9 for those born after 1984. Notably, among male ever-smokers, over 40% initiated regular smoking at an age younger than 20. A study conducted in South-West China (47) also reported that the older teens and young adults aged 15–19 were more likely to initiate cigarette smoking than other age groups. An updated data showed that ~90% of adult smokers in the United States used their first cigarette by 18 years of age (48). This is an alert for the importance of tobacco use prevention programs among adolescents. A qualitative study among Chinese male teenagers suggested that facilitating social interaction was the main reason for both cigarette initiation and acceptance (49). Additionally, based on the Population Assessment of Tobacco and Health (PATH) study (48), the use of e-cigarettes, hookah, non-cigarette combustible tobacco, or smokeless tobacco was associated with cigarette smoking. The increasing popularity of non-cigarette tobacco product among young people may contribute to the early initiation of cigarette smoking (50).

The present study revealed a high prevalence of smoking in male participants. However, unfortunately, the majority of smokers in China do not intend to quit smoking (51). Even in ever-diagnosed chronic disease patients, their current smoking prevalence was high: 43.83% in HTN patients, 41.50% in diabetes patients, and 37.87% in people with CVD. Smokers who had long time smoke duration appeared to have a lower likelihood of changing their smoking status (23). Male current smokers with HTN or other diseases in the present study had an average smoking duration over 30 years. For those younger smokers who had relatively shorter smoking duration, although they were diagnosed with chronic disease such as HTN, the lack of symptoms may be barriers for behavior change. The high prevalence of current smoking among people suffering from these diseases implied a suboptimal intervention effect to decrease health risk among the patients.

Although, in China, great efforts have been made in health care settings to provide health education and promotion on tobacco control for people who had chronic disease, considering the alarming high prevalence of current smoking revealed by this study, integrated strategies, such as psychological plus drugs intervention (17), and more emphasis should be given to reduce the health risk in this population.

Determining factors that influence smoking cessation may provide clues for tobacco control. As revealed by this study, the most influential factors of smoking cessation included ever being diagnosed with chronic disease (HTN and CVD), had shorter smoking duration, fewer cigarettes consumed per day, aging, and lower educational level. It is easy to understand that people who smoked for a long time may find it difficult to change their habit, and previous studies revealed similar findings (21, 22). On the other side, the relationships among smoking dose, duration, and smoking cessation may also be influenced by the reverse causality: poor health might affect smoking behavior. Although HTN, diabetes, and CVD had been adjusted in the multivariable regression model, the lack of a more comprehensive health assessment on the participants made it difficult to clarify this relationship, which was one of the study's limitations.

Awareness of the harms of smoking, or beliefs in health benefit due to smoking cessation, is associated with making a quit attempt (52, 53). FCTC has a key aspect to educate the public on the deleterious effects of tobacco (14). However, Chinese smokers have underestimated the health risk caused by smoking (54, 55). For people who were aware of their chronic disease status, they may benefit from the professional advice (such as seeking help in smoking cessation clinics) at medical examinations or health check-ups, which could be a motivator to stop smoking (56). A large prospective study in China supported that people who stopped smoking because of illness had higher prevalence of CVD, HTN, and diabetes than people who stopped by choice (57). In this study, educational attainment was found to be associated with smoking cessation. The possible reason may lie in the disparities of psychological or social–cultural factors that may play an important role in successful smoking cessation (58). This association still needs to be further investigated by longitudinal studies.

Aging could result in increased adverse conditions, weakness, and other health issues, which may become important motivators for smoking cessation (22). Consistent with other studies (57, 58), the predominant reason for quitting successfully was a detrimental health problem. For those who quit smoking because of illness, the suffering of respiratory disease was the predominant reason. The relationship between smoking and respiratory disease, such as COPD, is strong (59), and the symptoms of respiratory disease are obvious and thus could significantly affect patients' life quality. CVD ranked second in the reasons specified for smoking cessation. It may result from CVD's fatal outcome and severe symptoms. Other reasons for smoking cessation included realization that smoking presented a great health hazard, such as cerebrovascular disease and cancer, and with symptoms that affected individual's quality of life, such as pharyngitis or digestive system disease. This suggested that it may be effective to provide smoking cessation advice to people seeking health service, especially at primary care settings, such as community health centers in urban areas, or township hospitals in rural areas, where smoking screening and cessation counseling service could be integrated into health practitioners' routine work.

The proportion of smoking cessation by choice was around 50% in this study, higher than that in Liu et al.'s study (39.3%) (57). For men who quit smoking <5 years, especially those who quit by choice, there still are chances of relapsing, resulting in overestimation of the total percentage of quitters. Compared with never-smokers, there was no additional risk of diabetes for ex-smokers who quit by choice (57). Irrespective of whether this risk disparity due to smoking cessation reason is also the case in other diseases, it is important to initiate tobacco use interventions before disease onset.

There are some limitations in this study. First, since we used a cross-sectional design, we were not able to assess smoking relapse, especially for those who quit <2 years prior to the study. Limited by the questionnaire interview time, it was impractical for us to collect psychological and social environmental factors on smoking intention and cessation, which were important factors for smoking intervention (24, 51). We will focus on these factors in later studies. Second, smoking pattern, alcohol consumption, and chronic disease status were based on self-report, which may not reflect the true situation of participants' health-related behavior or status, and then led to misclassification bias. Third, the sample size of female participants was not enough, and the present study was conducted only in one province in China, resulted in limited external validity.

## Conclusions

This study revealed a high prevalence of smoking, especially in men, in a representative population in central China, and provided updated evidence of smoking patterns. Lifestyle risk factors such as alcohol consumption is found to be associated with smoking. Health education and interventions focused on tobacco control could be integrated with alcohol consumption reduction to achieve additional benefit. The high current smoking prevalence in people with chronic diseases produces a great challenge for tobacco control efforts in China. Considering that the predominant reason of smoking cessation is related to health, tobacco cessation programs should be initiated in health care settings to provide more targeted smoking cessation advices. Educational programs should be initiated early among teenagers to dissuade youth from starting smoking during the susceptible period.

## Data Availability Statement

The datasets generated or analyzed for this study are available from the corresponding author on reasonable request.

## Ethics Statement

The studies involving human participants were reviewed and approved by the institutional review board of Institute of Basic Medical Sciences, Chinese Academy of Medical Sciences. The patients/participants provided their written informed consent to participate in this study.

## Author Contributions

GS and HH: conceptualization, methodology, validation, writing–review and editing, and funding acquisition. HH: software, formal analysis, data curation, writing–original draft preparation, visualization. GS, LP, ZC, JS, CY, YC, and YW: investigation. GS and ZC: resources and supervision. GS and LP: project administration. All authors agreed to be accountable for the content of the work.

## Conflict of Interest

The authors declare that the research was conducted in the absence of any commercial or financial relationships that could be construed as a potential conflict of interest.
