# Supplementary material for: Smoking Prevalence, Patterns, and Cessation Among Adults in Hebei Province, Central China: Implications From China National Health Survey (CNHS)
Source: Front Public Health. 2020 Jun 11;8:177. doi: 10.3389/fpubh.2020.00177 (PMC7300263; doi:10.3389/fpubh.2020.00177)
Supplement: Supplementary file 1 [file Data_Sheet_1.docx]

Supplemental materials

**Table S1.** Smoking patterns, stratified by smoking status among female subjects in Hebei Province, central China, 2017.

|  | Ever smoker (n=148) | | Current smoker (n=116) | | Ex-smoker  (n=32) | | Cessation because of illness (n=20) | | Cessation by choice (n=12) | |
| --- | --- | --- | --- | --- | --- | --- | --- | --- | --- | --- |
| **Smoking started age**  (year, mean, SD) | 27.99 | 10.31 | 28.56 | 10.70 | 25.97 | 8.65 | 25.00 | 7.91 | 26.67 | 10.09 |
| categories (n, %) |  |  |  |  |  |  |  |  |  |  |
| <15 | 3 | 2.03 | 2 | 1.72 | 1 | 3.13 | 1 | 5.00 | 0 | 0 |
| 15- | 28 | 18.92 | 23 | 19.83 | 5 | 15.63 | 3 | 15.00 | 2 | 16.67 |
| 20- | 50 | 33.78 | 36 | 31.03 | 14 | 43.75 | 8 | 40.00 | 6 | 50.00 |
| 30- | 66 | 44.59 | 54 | 46.55 | 12 | 37.50 | 8 | 40.00 | 4 | 33.33 |
| **Smoking duration**  (years, mean, SD) | 24.83 | 16.06 | 26.31 | 15.92 | 19.50 | 15.66 ^a^ | 25.15 | 15.52 | 10.08 | 10.94 |
| Categories (n, %) |  |  |  |  |  |  |  |  |  |  |
| <5 | 19 | 12.84 | 10 | 8.62 | 9 | 28.13^b^ | 3 | 15.00 | 6 | 50.00 |
| 5- | 14 | 9.46 | 12 | 10.34 | 2 | 6.25 | 1 | 5.00 | 1 | 8.33 |
| 10- | 24 | 16.22 | 17 | 14.66 | 7 | 21.88 | 4 | 20.00 | 3 | 25.00 |
| 20- | 28 | 18.92 | 26 | 22.41 | 2 | 6.25 | 2 | 10.00 | 0 | 0 |
| 30- | 62 | 41.89 | 50 | 43.10 | 12 | 37.50 | 10 | 50.00 | 2 | 16.67 |
| **Daily cigarette**  **consumption** (mean, SD) | 12.19 | 9.32 | 12.29 | 9.11 | 11.84 | 10.14 | 14.95 | 11.05 | 6.67 | 5.68 |
| Categories (n, %) |  |  |  |  |  |  |  |  |  |  |
| ≤10 | 93 | 62.84 | 72 | 62.07 | 21 | 65.63 | 11 | 55.00 | 10 | 83.33 |
| 11- | 47 | 31.76 | 37 | 31.90 | 10 | 31.25 | 8 | 40.00 | 2 | 16.67 |
| >20 | 7 | 4.73 | 6 | 5.17 | 1 | 3.13 | 1 | 5.00 | 0 | 0 |
| **Duration from quit smoking to current time** (years, mean, SD) | NA | NA | NA | NA | 14.38 | 13.80 | 12.75 | 14.33 | 17.08 | 13.01 |
| Categories (n, %) |  |  |  |  |  |  |  |  |  |  |
| <2 | NA | NA | NA | NA | 4 | 12.50 | 4 | 20.00 | 0 | 0 |
| 2- | NA | NA | NA | NA | 8 | 25.00 | 6 | 30.00 | 2 | 16.67 |
| 5- | NA | NA | NA | NA | 2 | 6.25 | 1 | 5.00 | 1 | 8.33 |
| ≥10 | NA | NA | NA | NA | 18 | 56.25 | 9 | 45.00 | 9 | 75.00 |

a: *p* < 0.05 of the comparison between current-smoker and quit-smoker.

**Table S2.** The current smoking prevalence among participants with ever-diagnosed disease, stratified by sex, in Hebei Province, central China, 2017

|  | Male | | | | | | Female^b^ | | | | | |
| --- | --- | --- | --- | --- | --- | --- | --- | --- | --- | --- | --- | --- |
|  | HTN | | Diabetes | | CVD | | HTN | | Diabetes | | CVD | |
|  | n | %^a^ | n | % | n | % | n | %^a^ | n | % | n | % |
| **Total** | 391 | 43.83 | 105 | 41.50 | 103 | 37.87 | 48 | 4.29 | 10 | 3.51 | 25 | 5.27 |
| **Age groups** |  |  |  |  |  |  |  |  |  |  |  |  |
| 20- | 58 | 62.37 | 4 | 21.05 | 9 | 50.00 | 2 | 4.35 | 1 | 5.00 | 3 | 8.57 |
| 40- | 67 | 40.12 | 25 | 46.30 | 13 | 50.00 | 5 | 2.48 | 1 | 2.33 | 0 | 0 |
| 50- | 148 | 47.13 | 48 | 50.53 | 42 | 50.60 | 17 | 4.03 | 5 | 5.15 | 9 | 5.49 |
| 60-80 | 118 | 37.11 | 28 | 32.94 | 39 | 26.90 | 24 | 5.33 | 3 | 2.40 | 13 | 6.05 |
| **Resident areas** |  |  |  |  |  |  |  |  |  |  |  |  |
| Urban | 140 | 40.11 | 44 | 37.29 | 43 | 41.35 | 2 | 0.51 | 1 | 0.98 | 1 | 0.59 |
| Rural | 251 | 46.22 | 61 | 45.19 | 60 | 35.93 | 45 | 6.17 | 9 | 4.92 | 24 | 7.95 |
| **Education** |  |  |  |  |  |  |  |  |  |  |  |  |
| Primary school or lower | 97 | 44.91 | 18 | 43.90 | 30 | 37.50 | 40 | 6.60 | 7 | 4.38 | 21 | 7.61 |
| High school | 243 | 45.68 | 66 | 43.71 | 60 | 37.50 | 7 | 1.58 | 2 | 1.79 | 4 | 2.44 |
| College or higher | 51 | 35.66 | 21 | 34.43 | 13 | 40.63 | 0 | 0 | 0 | 0 | 0 | 0 |
| **Alcohol drinking** | | |  |  |  |  |  |  |  |  |  |  |
| Never drinking | 33 | 36.67 | 12 | 32.43 | 13 | 36.11 | 32 | 3.76 | 8 | 3.56 | 16 | 4.46 |
| Quit drinking | 43 | 32.09 | 13 | 39.39 | 17 | 29.82 | 3 | 4.55 | 1 | 6.67 | 1 | 3.57 |
| Current drinking | 315 | 47.16 | 80 | 43.72 | 73 | 40.78 | 13 | 6.44 | 1 | 2.22 | 8 | 9.20 |
| **Alcohol consumption (g/day)** | | |  |  |  |  |  |  |  |  |  |  |
| Never drinking | 33 | 36.67 | 12 | 32.43 | 13 | 36.11 | 32 | 3.76 | 8 | 3.56 | 16 | 4.46 |
| < 40 | 213 | 42.86 | 51 | 38.06 | 54 | 34.84 | 12 | 4.88 | 1 | 1.82 | 5 | 5.00 |
| 41-60 | 47 | 43.93 | 13 | 50.00 | 13 | 44.83 | 2 | 15.38 | 0 | 0 | 3 | 30.00 |
| > 60 | 98 | 49.49 | 29 | 51.79 | 23 | 44.23 | 2 | 25.00 | 1 | 33.33 | 1 | 20.00 |

a: the prevalence of current smoking within each stratum among those who had diagnosed diseases. b: Statistical test was not applied because of limited sample size in each cell.

**Table S3**. The associated factors of ever smoking in male participants, stratified by residential areas, in Hebei Province, central China, 2017.

|  | **Urban** | | | | | | **Rural** | | | | | |
| --- | --- | --- | --- | --- | --- | --- | --- | --- | --- | --- | --- | --- |
|  | n | % | *OR* | *95% CI* | | *p* | n | % | *OR* | *95% CI* | | *p* |
| **Age (every ten years)** | NA | NA | 1.04 | 0.93 | 1.16 | 0.531 | NA | NA | 1.06 | 0.95 | 1.17 | 0.302 |
| **Education attainment** |  |  |  |  |  |  |  |  |  |  |  |  |
| Primary school  or lower | 68 | 74.73 |  | 2.41 | 1.33 | 0.004 | 382 | 80.76 | 4.17 | 2.46 | 7.08 | <0.001 |
| High school | 381 | 67.43 |  | 1.79 | 1.30 | <0.001 | 643 | 69.74 | 2.41 | 1.51 | 3.84 | <0.001 |
| College or higher | 225 | 51.96 | 1 | NA | NA | NA | 49 | 45.37 | 1 | NA | NA | NA |
| **Occupation** |  |  |  |  |  |  |  |  |  |  |  |  |
| Farmer | 5 | 62.50 | 1 | NA | NA | NA | 291 | 75.19 | 1 | NA | NA | NA |
| Factory worker |  |  | 2.60 | 0.54 | 12.57 | 0.235 | 50 | 70.42 | 0.99 | 0.52 | 1.89 | 0.969 |
| Technician | 70 | 77.78 | 1.47 | 0.32 | 6.83 | 0.622 | 71 | 56.35 | 0.88 | 0.53 | 1.46 | 0.612 |
| Officer | 153 | 55.84 | 1.09 | 0.23 | 5.17 | 0.918 | 9 | 56.25 | 0.78 | 0.26 | 2.30 | 0.649 |
| Unemployed | 59 | 50.43 | 0.58 | 0.11 | 3.02 | 0.521 | 101 | 79.53 | 1.25 | 0.76 | 2.06 | 0.390 |
| Others | 15 | 50.00 | 1.61 | 0.36 | 7.26 | 0.533 | 552 | 71.13 | 1.02 | 0.74 | 1.42 | 0.887 |
| **Personal income (RMB per year)** ^a^ | | |  |  |  |  |  |  |  |  |  |  |
| < 10000 | 42 | 63.64 | 1 | NA | NA | NA | 401 | 76.38 | 1 | NA | NA | NA |
| 10000- | 152 | 65.52 | 0.95 | 0.51 | 1.77 | 0.866 | 378 | 72.69 | 0.98 | 0.71 | 1.34 | 0.895 |
| 30000- | 297 | 60.24 | 0.88 | 0.47 | 1.64 | 0.681 | 163 | 62.45 | 0.82 | 0.55 | 1.22 | 0.329 |
| ≥ 50000 | 183 | 61.20 | 1.03 | 0.54 | 1.97 | 0.930 | 128 | 66.67 | 1.03 | 0.66 | 1.62 | 0.898 |
| **Alcohol consumption (g/day)** | |  |  |  |  |  |  |  |  |  |  |  |
| Never drinking | 67 | 58.26 | 1 | NA | NA | NA | 113 | 59.47 | 1 | NA | NA | NA |
| < 40 | 410 | 58.74 | 1.16 | 0.77 | 1.76 | 0.477 | 606 | 69.10 | 1.70 | 1.21 | 2.37 | 0.002 |
| 41-60 | 87 | 73.11 | 2.37 | 1.33 | 4.21 | 0.003 | 128 | 83.12 | 3.48 | 2.06 | 5.86 | <0.001 |
| > 60 | 110 | 69.62 | 1.82 | 1.08 | 3.07 | 0.024 | 227 | 80.50 | 2.99 | 1.96 | 4.58 | <0.001 |

^a^1RMB≈0.14USD

**Table S4**. Reasons for smoking cessation among male subjects.

| **Cessation because of illness** | n | % |
| --- | --- | --- |
| **Total** | 244 | 50.94 |
| Respiratory disease | 94 | 38.52 |
| Cardiovascular disease | 48 | 19.67 |
| Other disease | 28 | 11.48 |
| Pharyngitis | 25 | 10.25 |
| Cerebrovascular disease | 17 | 6.97 |
| Digestive system disease | 15 | 6.15 |
| Ear and nose disease | 5 | 2.05 |
| Bone and joint disease | 4 | 1.64 |
| Oral health issue | 4 | 1.64 |
| Cold | 3 | 1.23 |
| Cancer | 1 | 0.41 |
| **Cessation by choice** |  |  |
| **Total** | 235 | 49.06 |
| Others | 227 | 96.60 |
| Pregnancy | 3 | 1.28 |
| Economic pressure | 3 | 1.28 |
| Military service | 2 | 0.85 |

**Table S5**. Demographic characteristics of ex-smokers among male participants in Hebei Province, China, 2017.

| Characteristics | Ex-smoker (N=483) | | Cessation because of illness (n=244) | | Cessation by choice (n=235) | |
| --- | --- | --- | --- | --- | --- | --- |
|  | n | % | n | % | n | % |
| **Age group** |  |  |  |  |  |  |
| 20- | 46 | 9.52 | 12 | 4.92 | 32 | 13.62^bc^ |
| 40- | 224 | 46.38 | 105 | 43.03 | 118 | 50.21 |
| 60-80 | 213 | 44.10 | 127 | 52.05 | 85 | 36.17 |
| **Marital status** |  |  |  |  |  |  |
| Unmarried | 5 | 1.04 | 2 | 0.82 | 3 | 1.28 |
| Married | 461 | 95.45 | 235 | 96.31 | 222 | 94.47 |
| Divorced or widowed | 17 | 3.52 | 7 | 2.87 | 10 | 4.26 |
| **Resident area** |  |  |  |  |  |  |
| Urban | 190 | 39.34 | 85 | 34.84 | 103 | 43.83^b^ |
| rural | 293 | 60.66 | 159 | 65.16 | 132 | 56.17 |
| **Education attainment** |  |  |  |  |  |  |
| Primary school or lower | 144 | 29.81 | 78 | 31.97 | 65 | 27.66 |
| High school | 274 | 56.73 | 135 | 55.33 | 137 | 58.30 |
| College or higher | 65 | 13.46 | 31 | 12.70 | 33 | 14.04 |
| **Occupation** |  |  |  |  |  |  |
| Farmer | 89 | 18.43 | 48 | 19.67 | 40 | 17.02 |
| Factory worker | 40 | 8.28 | 24 | 9.84 | 16 | 6.81 |
| Technician | 67 | 13.87 | 32 | 13.11 | 35 | 14.89 |
| Officer | 21 | 4.35 | 10 | 4.10 | 11 | 4.68 |
| No job | 51 | 10.56 | 27 | 11.07 | 24 | 10.21 |
| Others | 215 | 44.51 | 103 | 42.21 | 109 | 46.38 |
| **Personal income (RMB/year)** | |  |  |  |  |  |
| <10000 | 133 | 27.54 | 69 | 28.28 | 64 | 27.23 |
| 10000- | 136 | 28.16 | 69 | 28.28 | 67 | 28.51 |
| 30000- | 133 | 27.54 | 66 | 27.05 | 64 | 27.23 |
| ≥50000 | 80 | 16.56 | 40 | 16.39 | 39 | 16.60 |
| **Alcohol drinking** |  |  |  |  |  |  |
| Never drinking | 39 | 8.07 | 23 | 9.43 | 16 | 6.81^b^ |
| Quit drinking | 100 | 20.70 | 62 | 25.41 | 37 | 15.74 |
| Current drinking | 344 | 71.22 | 159 | 65.16 | 182 | 77.45 |
| **Alcohol consumption** |  |  |  |  |  |  |
| Never drinking | 39 | 8.07 | 23 | 9.43 | 16 | 6.81 |
| Low consumption | 289 | 59.83 | 148 | 60.66 | 138 | 58.72 |
| Moderate consumption | 63 | 13.04 | 26 | 10.66 | 37 | 15.74 |
| High consumption | 92 | 19.05 | 47 | 19.26 | 44 | 18.72 |

a: the sum of quit smoking because of illness and quit smoking by choice was not equal to 483 because of there were four missing values on the reason of smoking cessation; b: *p* < 0.05 of the comparison between quit smoking because of illness and quit smoking by choice; c: *p* < 0.01 for the Cochran-Armitage trend test.
